# Mortality at the North and at the South

**Published:** 1855-01

**Authors:** 


Mortality at the North and at the South.—The experience of the Life Insurance Companies of this country has brought to light certain facts which are not expressed or indicated by the census returns. In reference to the North, or that portion of the Union embraced northerly of the southern line of Virginia and Kentucky, it is shown that the mortality is considerably less than that indicated by the celebrated Carlisle tables. From ten years' practical results, it appears that the mortality among that class of persons who insure their lives within the region named is less than 1 per cent., viz: about 94 in every 10,000. In that section comprised between 32° north latitude (a few miles south of Savannah) and the southern line of Virginia and Kentucky, the mortality among the same class of people is 1. 17 1-4 per cent., or 117 1-4 persons out of every 10,000. Looking to the extreme Southern States, or south of latitude 32°, the mortality is nearly double that of the Northern section—the actual number of deaths of 10,000 (as insured) being 186. These calculations are entirely exclusive of the present year, which has perhaps been more fatal in the South than any period since 1830.—*Louisville Journal.*

